# Balancing selectivity retention and catalyst restructuring in pulsed CO_2_ electroreduction on silver

**DOI:** 10.1039/d6ey00140h

**Published:** 2026-08-21

**Authors:** Claudia Bissattini Alessi, Richa Rajadhyax, Alessandro Senocrate, Corsin Battaglia

**Affiliations:** a Empa – Swiss Federal Laboratories for Materials Science and Technology, Materials for Energy Conversion CH-8600 Duebendorf Switzerland alessandro.senocrate@empa.ch; b Institute of Materials, École Polytechnique Fédérale de Lausanne CH-1015 Lausanne Switzerland; c Department of Information Technology and Electrical Engineering, ETH Zurich CH-8092 Zurich Switzerland

## Abstract

Pulsed electrochemical CO_2_ reduction has emerged as a strategy to extend electrolyzer lifetime by periodically interrupting reductive operation with short oxidative pulses. Here we investigate the effect of oxidative pulse potential, pulse frequency, and pulse duration on the selectivity and structural integrity of electrospun polymer-based silver gas diffusion electrodes over extended periods of operation. We show that the threshold potential for anodic dissolution of silver at 0.55 V *vs.* SHE defines an upper boundary for the pulse potential: pulses with a potential below this threshold enable periodic local acidification of the electrolyte in the vicinity of the catalyst promoting partial dissolution of (bi)carbonate precipitates, while pulses with a potential above the threshold drive a silver dissolution-redeposition cycle that restructures the catalyst into silver clusters promoting the hydrogen evolution reaction. Systematic exploration of the pulse parameter space identifies an optimal protocol consisting of 10-seconds-long oxidative pulses applied every 30 minutes, yielding a duty cycle exceeding 99%. Under these conditions, carbon product faradaic efficiency is maintained in the range of 70–80% over 100 hours of operation, representing a five-fold improvement in selectivity relative to continuous electrolysis. Our results demonstrate the potential of pulsed electrolysis to maintain high CO selectivity over long operational periods, thus representing an important step towards the industrialization of CO_2_ electrolysis.

## Abstract 2

Oxidative pulses above the silver dissolution threshold cause irreversible catalyst restructuring; pulses below it sustain CO selectivity over 100 hours.

## Graphical abstract

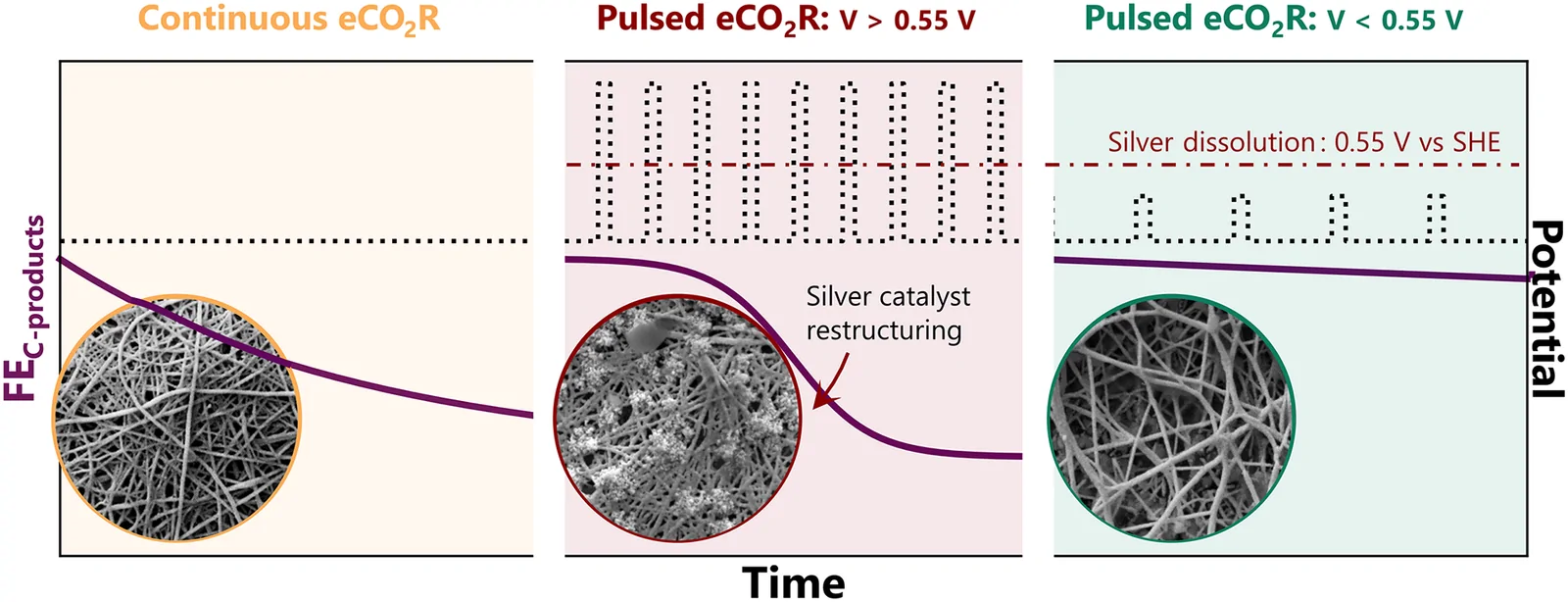

**Broader context**

Carbon monoxide is an important industrial feedstock: together with hydrogen, it forms synthesis gas, the basis for the Fischer–Tropsch synthesis of a wide range of fuels and chemicals. Producing CO through electrochemical CO_2_ reduction using renewable electricity offers a route to close the carbon cycle while replacing energy-intensive thermal processes. Silver is the leading catalyst for this reduction reaction, as it can achieve high CO selectivity at industrially relevant current densities. However, CO selectivity rapidly degrades over time due to bicarbonate salt precipitation caused by the reaction shifting the local pH to alkaline. This degradation can be mitigated using pulsed operation, where potential pulses periodically interrupt the electrochemical CO_2_ reduction. Pulsing improves electrolyzer lifetime, but the choice of pulse potential has consequences for silver catalyst morphology that have yet to be thoroughly studied. In this work, we show that the silver dissolution potential separates two fundamentally different outcomes: pulses below this threshold restore the catalyst's local pH thus dissolving accumulated salt deposits, sustaining CO selectivity over 100 hours; pulses above it trigger irreversible catalyst restructuring that accelerates failure. These findings provide a mechanistic basis for the design of pulse protocols for durable CO_2_ electrolyzers.

## 1. Introduction

The electrochemical reduction of carbon dioxide (eCO_2_R) represents a promising technology to convert CO_2_ into valuable fuels and platform chemicals with electricity sourced from renewable energy.^1–5^ To address the low solubility of CO_2_ in aqueous media, gas diffusion electrodes (GDEs) are employed to deliver CO_2_ directly to the catalyst surface, thereby enabling operation at high current densities.^6^ Despite rapid progress, long-term product selectivity retention remains a critical bottleneck, with performance degradation arising from several phenomena, including catalyst restructuring, (bi)carbonate salt precipitation, and GDE flooding by the electrolyte.^7–9^

Pulsed eCO_2_R, in which the reductive potential required for CO_2_ conversion is periodically interrupted by short pulses at an oxidative, or less reducing, potential, has emerged as a practical strategy to counteract these degradation phenomena and extend electrolyzer lifetime.^10,11^ Pulsing was demonstrated to restore catalyst activity, delay deactivation, and in some cases shift product selectivity, with reported significant lifetime improvements.^12,13^ The beneficial effects of pulsing have been attributed to different mechanisms according to their timescale: millisecond pulses modulate adsorbate populations and double-layer composition, seconds-long pulses influence local diffusion gradients and the distribution of cations, while minutes-long pulses are thought to address slower phenomena such as GDE flooding, and removal of liquid byproducts.^14–20^ In principle, a protocol with short and infrequent pulses would be energetically attractive, since each pulse interrupts productive CO_2_ reduction and introduces non-faradaic losses from recharging the electrochemical double layer.^21,22^

Unlike copper, whose complex product distribution and propensity for restructuring have motivated the majority of pulsed eCO_2_R studies, silver offers a simpler product distribution, being primarily selective towards CO with high faradaic efficiency (FE), with additional contributions only from hydrogen and formic acid.^23,24^ Pulsed electrolysis has nevertheless been shown to further enhance the performance of silver catalysts by improving CO selectivity and stability.^11,12,14–20,23^ However, the structural consequences of pulsed eCO_2_R on silver catalysts have, to our knowledge, not yet been investigated, despite their high sensitivity to electrochemically-induced morphological restructuring.^25–29^ Here we address this gap by investigating pulsed eCO_2_R on polymer-based silver GDEs and exploring the effect of pulse potential, pulse frequency, and pulse duration on selectivity and catalyst morphology. We show that the threshold potential for anodic silver dissolution defines a boundary between beneficial and detrimental pulsing regimes. Oxidative pulses below this threshold regenerate the catalyst microenvironment while preserving catalyst morphology, whereas pulses above it trigger irreversible catalyst restructuring and accelerated selectivity loss. Within this mechanistic framework, we identify an optimal pulsing protocol that combines infrequent 10 s oxidative pulses with a >99% duty cycle resulting in stable CO selectivity over 100 h of operation.

## 2. Experimental

GDEs with silver as catalyst were prepared by DC magnetron sputtering (AJA International Orion) of ∼400 nm silver catalyst layer (sputtering time 10 min, power 5 W m^−2^, argon pressure 0.004 mbar, deposition rate ∼0.6 nm s^−1^ as measured by quartz microbalance (Inficon)) onto one side of an electrospun hydrophobic porous polymer substrate. Polymer gas diffusion layers (GDLs) with a mean pore size of 200 nm were prepared by electrospinning a 15 wt% solution of polyvinylidene fluoride-*co*-hexafluoropropylene (PVDF-HFP, Merck, Mw ∼ 400 k, Mn ∼ 130 k) with 1 wt% tetraethylammonium bromide (TEABr, Merck, 98%) in dimethylformamide (DMF, Merck, 227056, 99.8%) at 30 kV, a solution flow rate of 10 µL min^−1^, collector drum rotation of 400 rpm, and a needle-to-collector distance of 13 cm.^30^

CO_2_ electrolysis was performed in a three-compartment polyether ether ketone (PEEK) electrochemical cell (Redox.me) with an active area of 1 cm^2^ with separate gas, catholyte, and anolyte chambers, schematized in Fig. S1a. The GDE was mounted as the cathode with its hydrophobic polymer side facing the CO_2_ gas stream and the silver-coated side in contact with the catholyte. An IrO_*x*_ catalyst electrodeposited onto a titanium mesh served as anode, and an Ag/AgCl electrode (filled with saturated KCl) as reference. Catholyte and anolyte chambers were separated by a perfluorosulfonic acid-based cation exchange membrane (FuelCellStore PFSA D50-U). All experiments used 1 M KHCO_3_ (99.7%, Merck) as catholyte, pre-saturated with CO_2_ by bubbling prior to electrolysis, and 1 M H_2_SO_4_ (95–97%, Merck) as anolyte. The 1 M H_2_SO_4_ anolyte was selected instead of *e.g.* KHCO_3_ to avoid continuous transport of K^+^ from anode to cathode, which leads to conductivity changes, pH imbalances, and faster (bi)carbonate precipitation at the cathode side. Moreover, H_2_SO_4_ reduces the overpotential of the oxygen evolution reaction on IrO_*x*_. Although a continuous proton flux from the anolyte to the catholyte can be expected under OCV, this is minor compared to the current-induced transport, as demonstrated by the catholyte pH changing from 7.8 to 7.4 after 10 hours and to 7.3 after 20 hours. For long-term experiments lasting 50 hours, the pH reaches only ∼7.2 due to the large catholyte volume and its buffering capacity. The CO_2_ flow to the gas compartment was set to 20 mL min^−1^ by a mass flow controller (Bronkhorst) and monitored by a mass flow meter (Defender 530+, MesaLabs). The electrochemical pulse protocol applied *via* a multi-channel potentiostat (Biologic SP300) varied between the different experiments, therefore the specific conditions will be described in the results and discussion section. Products were quantified using gas chromatography (Inficon MicroGC Fusion) and liquid chromatography (Agilent HPLC 1260) as described in our previous work (details in Supporting notes 1 and 2).^31^ The silver catalyst morphology after electrolysis was imaged using scanning electron microscopy (SEM) (Thermo Fisher Phenom). Cross sections were prepared and imaged by focused ion beam SEM using a gallium ion beam at 30 kV for milling, with secondary electron imaging at 2 kV (Zeiss CrossBeam 540). Inductively coupled plasma mass spectrometry (Thermo Scientific iCAP Q) was employed to quantify Ag dissolution into the catholyte after electrolysis.

## 3. Results and discussion

We first explore the impact of pulse voltage on the product selectivity and stability of polymer-based Ag GDEs. Polymer substrates are chosen due to their high hydrophobicity and water entry pressure, eliminating macroscopic flooding as a degradation mechanism.

**Fig. 1:**
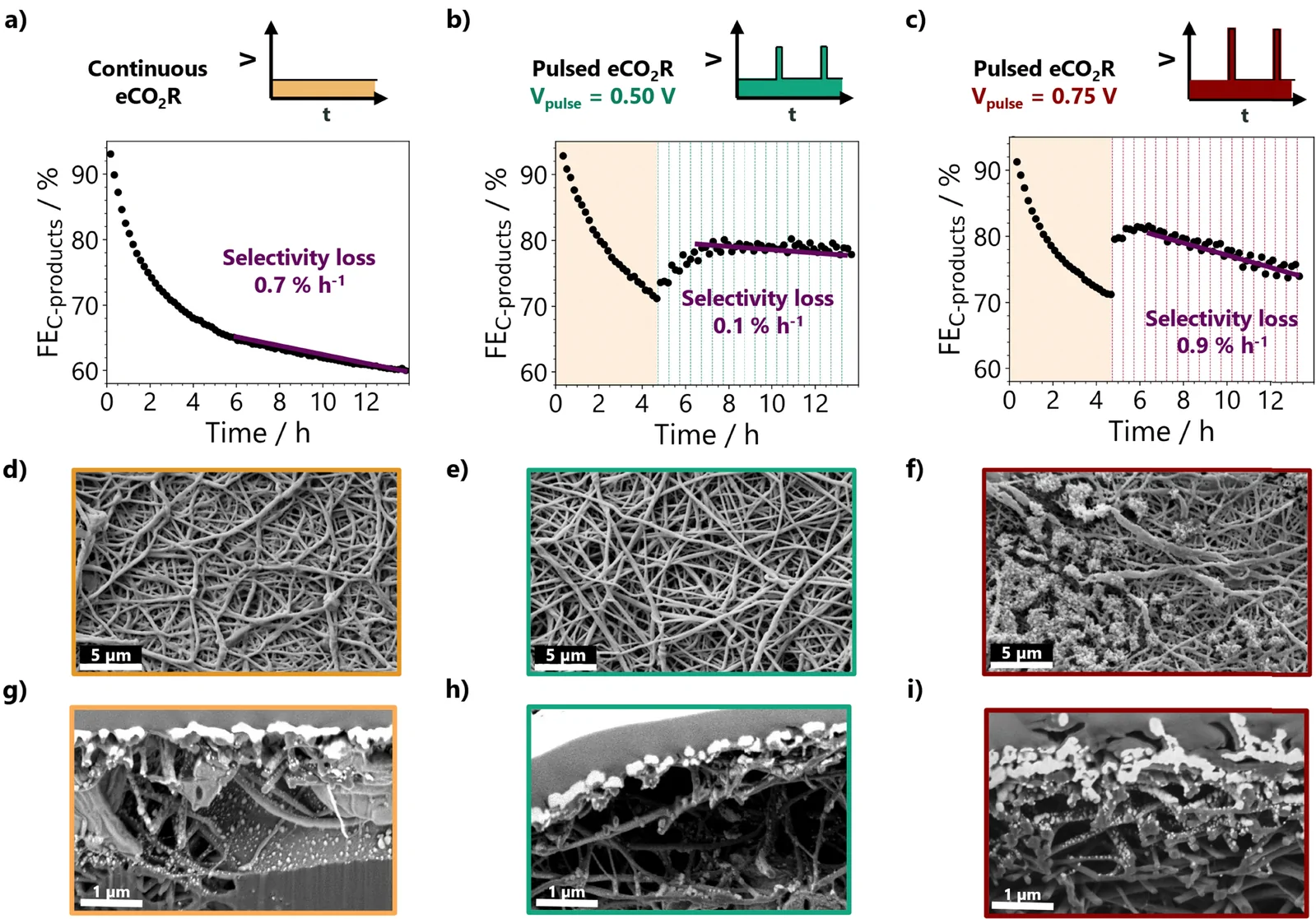
(a)–(c) Faradaic efficiency towards carbon products for a cell operated at 200 mA cm^−2^ (a) continuously for 13 hours, (b) continuously for 5 hours and then pulsed for 5 seconds at +0.5 V *vs.* SHE every 30 minutes for 8 hours, and (c) continuously for 5 hours and then pulsed for 5 seconds at +0.75 V *vs.* SHE every 30 minutes for 8 hours. (d)–(f) Top-view and (g)–(i) cross-section-view SEM images of the GDEs employed in the experiments in (a)–(c).

Fig. 1 shows the evolution of the FE towards carbon products (FE_C-products_ = 100% − FE_H_2__), namely CO and formic acid, of the silver GDE during continuous and pulsed electrolysis over 13 hours, alongside SEM images of the catalyst surface after operation. To compare continuous and pulsed experiments, we define the selectivity loss rate as the slope of a linear fit of the last 7 hours of the FE *vs.* time curves during which oxidative pulses were applied. Fig. 1a shows the continuous electrolysis experiment, where the selectivity decays steadily at an average rate of 0.6% h^−1^, falling from an initial value of approximately 91% to around 61% by the end of the experiment. This gradual decline is consistent with a rise in local pH, reducing the CO_2_ availability *via* speciation in the vicinity of the catalyst surface. This phenomenon also causes progressive (bi)carbonate salt precipitation within the GDE pore structure, further reducing CO_2_ access, and shifting selectivity toward HER. The occurrence of substantial (bi)carbonate precipitation in polymer GDEs operated in flow cells has been previously measured by our group using *operando* synchrotron wide-angle X-ray scattering^13^ (additional data shown in Fig. S13 and discussed in Note S5).

After an initial 5 hours of continuous operation, introducing 5-seconds-long oxidative pulses at 0.50 V *vs.* SHE every 30 minutes reduces the selectivity loss rate to just 0.1% h^−1^ as shown in Fig. 1b, representing a significant improvement in stability relative to continuous operation. The FE_C-products_, which drops to 72% after the initial 5 hours of continuous electrolysis, recovers partially and stabilizes at 78–80% until the end of the experiment, suggesting that the pulses periodically restore the conditions necessary for selective CO_2_ reduction. Interestingly, increasing the oxidative pulse potential to 0.75 V *vs.* SHE (Fig. 1c) produces the opposite effect, with the selectivity loss rate during the pulsed phase accelerating to 0.9% h^−1^ and significantly exceeding even that of continuous operation.

The SEM images in Fig. 1d–f provide a first structural explanation. After continuous electrolysis, the polymer fibers of the GDE remain uniformly coated with silver (Fig. 1d). The same occurs when pulsing at 0.5 V (Fig. 1e). However, increasing the pulse voltage to 0.75 introduces a severe restructuring of the silver catalyst in the form of micron-sized, irregular silver clusters decorating the fibers (Fig. 1f).

**Fig. 2:**
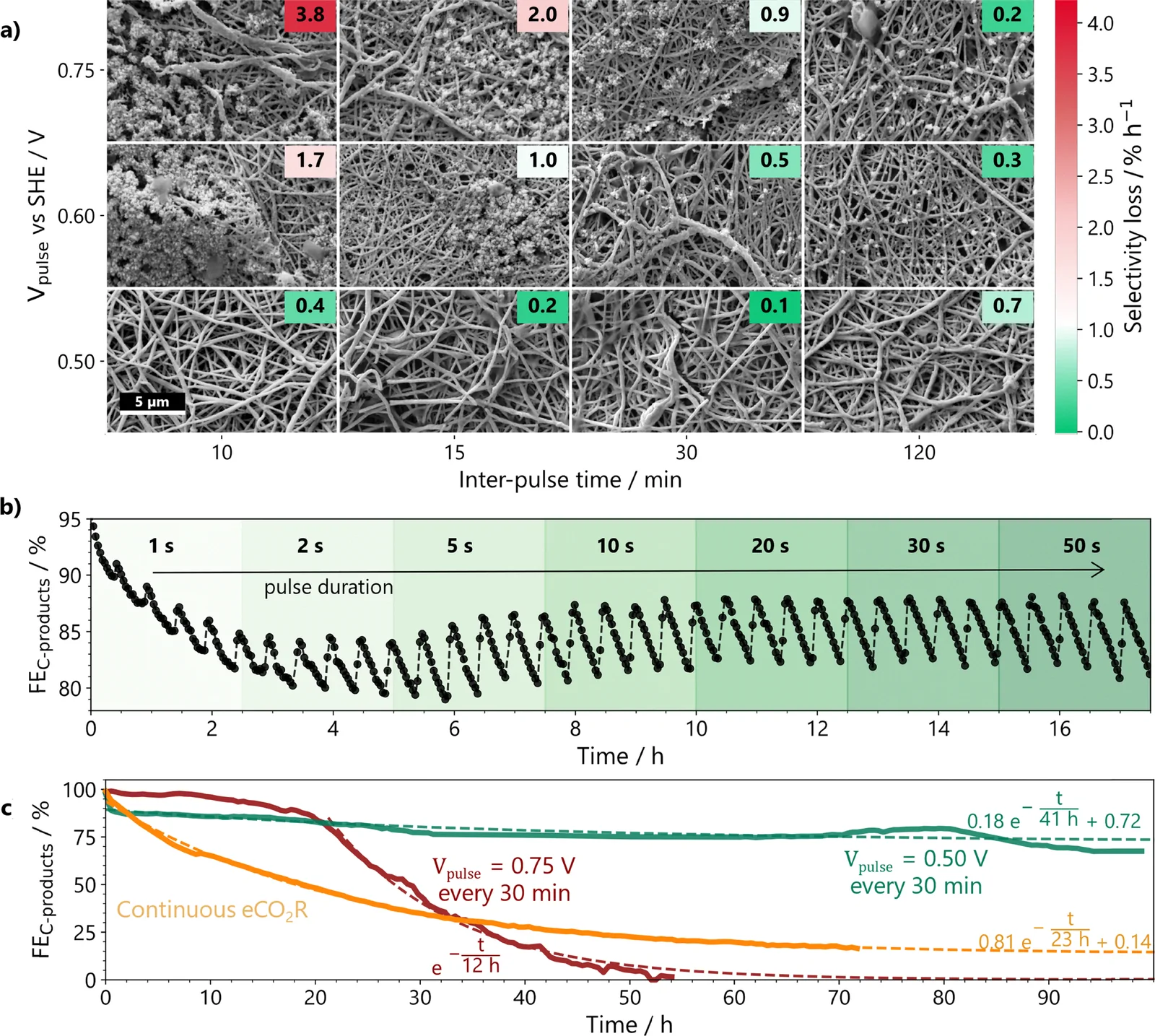
(a) Top-view *post mortem* SEM images of the silver GDE for different combinations of pulse potentials and time between pulses with the extracted selectivity loss value for each experiment. (b) Faradaic efficiency towards carbon products for different oxidative pulse durations with pulses applied every 30 minutes. (c) Faradaic efficiency towards carbon products for 100 hours of pulsed electrolysis with 0.50 V oxidative pulses for 10 seconds every 30 minutes of electrolysis at 200 mA cm^2^, compared to the evolution of the faradaic efficiency during continuous electrolysis and during pulsed electrolysis with 0.75 V oxidative pulses for 10 seconds every 30 minutes. Experimental curves are fitted with an exponential decay (from 20 hours onwards for the pulsed at 0.75 V) to compare the decay constant *τ* (41 hours, 23 hours, 12 hours) and the theoretical steady-state value (72%, 14%, 0%).

To better understand the causes of this behavior, we systematically explore the parameter space of pulse potential, pulse frequency, and pulse duration for Ag GDEs. Fig. 2a presents a matrix of *post mortem* SEM images and corresponding selectivity loss rates for 12 combinations of pulse potential (0.50, 0.60, and 0.75 V *vs.* SHE) and time between each pulse (10, 15, 30, and 120 min). The color scale represents the selectivity loss rate for the corresponding experiments, providing an immediate visual overview of the parameter space. Given the simplistic nature of the linear fit and some variability in the duration of the experiments (as can be seen in Fig. S2), we use this matrix to identify general trends and find a protocol that minimizes selectivity loss while maximizing the duty cycle. Along the vertical axis of Fig. 2a, the consequences of higher voltage pulses become evident across all pulse frequencies. At 0.75 V, the SEM images consistently show heavily aggregated silver clusters and the highest selectivity loss rates. At 0.60 V, intermediate morphologies are observed, with partial aggregation visible, particularly for shorter intervals between pulses, and selectivity loss rates that are reduced but remain significant. At 0.50 V, the catalyst morphology is best preserved across all pulse frequencies, with SEM images closely resembling the as deposited conformal silver layers, and selectivity loss rates that are consistently the lowest.

Along the horizontal axis, a clear and monotonic trend emerges at 0.60 and 0.75 V: shorter reductive periods between oxidative pulses yield higher selectivity loss rates and more disrupted catalyst morphologies, while longer intervals between pulses result in progressively lower degradation rates and better-preserved structures. In the case of the 0.50 V pulses, the shorter inter-pulse time appears to induce a faster loss in selectivity between 10 and 30 minutes. We attribute this difference to sample-to-sample variability as it falls within the error bars of our measurements (see Fig. S3 in Note S3) rather than to a systematic effect of the pulsing protocol. Notably, increasing the inter-pulse time further to 120 min at 0.50 V leads to a pronounced increase in selectivity loss rate to 0.7% h^−1^, suggesting that excessively long intervals between pulses partially negate the benefit of pulsing by approaching continuous operation, a behavior clearly visible in Fig. S2.

We then move to evaluate the effect of the duration of an oxidative pulse at 0.50 V *vs.* SHE on product selectivity. Fig. 2b shows the FE_C-products_ over approximately 17 hours as the pulse duration is stepped from 1 second up to 50 seconds at 30 minutes intervals. At the shortest pulse durations of 1 second and 2 seconds, the selectivity continues to decay between pulses, with only a marginal recovery visible immediately after each pulse, indicating that these pulse durations are insufficient to meaningfully restore the catalyst selectivity. As the pulse duration increases to 5 seconds, a progressively more pronounced recovery of FE_C-products_ is observed after each pulse. Beyond 10 s, the recovery per pulse appears to reach a plateau, with further increases in pulse duration to 20, 30 and 50 seconds yielding no additional improvement in FE_C-products_, which stabilizes at 85–87%. The characteristic timescale of several seconds provides insight into the underlying mechanism. Processes occurring at the electrode interface, such as double-layer relaxation and surface adsorbate removal, are generally expected to occur on sub-second timescales and therefore cannot explain the strong dependence on pulse duration between 1 and 10 s.^32^ Conversely, the absence of an additional benefit for pulses beyond 10 s argues against slower processes such as flooding mitigation.^20^ Additional experimental evidence and *operando* Raman spectroscopy data given in Note S5 (Fig. S10 and S11) confirm a local pH swing and suggests partial bicarbonate salt dissolution and increased CO_2_ availability as the main cause for selectivity recovery.

Having established that 0.50 V and 0.75 V pulses produce fundamentally different outcomes in GDE structures, we evaluate whether these differences compound or plateau over extended periods of operation. GDEs were operated for 100 hours under continuous electrolysis, 0.50 V pulsed, and 0.75 V pulsed conditions at 200 mA cm^−2^ with the latter two employing 10 s pulses every 30 minutes. The results are shown in Fig. 2c, where the data obtained by continuous and 0.50 V pulsed electrolysis are fitted with an exponential decay of the form *ṅ*_C-products_(*t*) = *ṅ*_0_·e^−*t*/*τ*^ + *ṅ*_∞_, where *ṅ* is the rate of formation of carbon products. In the plot, the fitting equation terms are converted to FE 
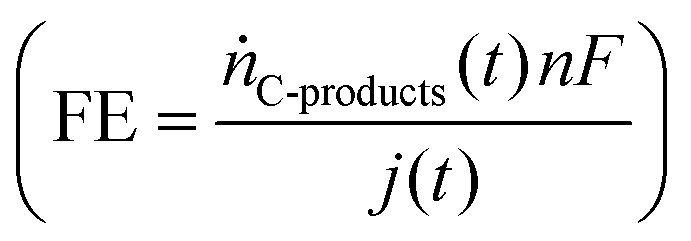
 for immediate understanding. The results of the experiment with pulses at 0.75 V are fitted only from 20 hours onwards, given the initially flat behavior. Under continuous electrolysis, the *ṅ*_C-products_ decays rapidly. A decay constant *τ* of 23 hours indicates that the electrode loses most of its selectivity within the first two days of operation resulting in a steady-state value of only 0.15 µmol s^−1^ (FE = 14%) implying near-complete loss of eCO_2_R selectivity in favor of HER. The rate of selectivity loss decreases over time towards a quasi-equilibrium state in which (bi)carbonate salt accumulation progressively blocks CO_2_ access to active catalyst sites, thereby reducing the eCO_2_R reaction rate and (bi)carbonate precipitation itself.

With 0.5 V pulsed electrolysis, the FE_C-products_ still undergoes an initial decay in the first 20–30 hours of operation, reflecting the onset of degradation processes that pulsing can slow down but not entirely prevent. However, the decay quickly flattens, and the selectivity approaches a stable plateau. The fitted decay constant nearly doubles relative to continuous operation, and most importantly, the steady state *ṅ*_C-products_ remains remarkably high (0.72–0.83 µmol s^−1^, FE = 70–80%), representing a five-fold improvement over the corresponding value for continuous electrolysis, and demonstrating that the pulsing protocol can sustain meaningful eCO_2_R performance over extended periods of time that are clearly unachievable under continuous operation.

While carrying out the experiments with a pulse potential of 0.75 V, the FE_C-products_ remains relatively high during the first 20 hours (corresponding to a selectivity loss rate of approximately 1% h^−1^, consistent with the data in Fig. 2a). However, the restructuring of the catalyst progressively leads to an accelerated decay towards 0% FE (*τ* = 12 hours). This behavior is mirrored by the working electrode potential and resistance during the 50 hours of operation, which drops from −4 V *vs.* SHE to −10 V *vs.* SHE and increases from 10 to 20 Ohms, respectively (Fig. S8b). As each pulse drives further restructuring, the catalyst layer becomes increasingly fragmented and protrudes towards the electrolyte. The resulting lack of local CO_2_ availability shifts selectivity towards HER, requiring progressively higher potentials. *Post mortem* inspection reveals that the area of the GDE exposed to the electrolyte has detached almost fully from the surrounding silver layer, as can be seen in Fig. S8a, consistent with the increasing resistance.

**Fig. 3:**
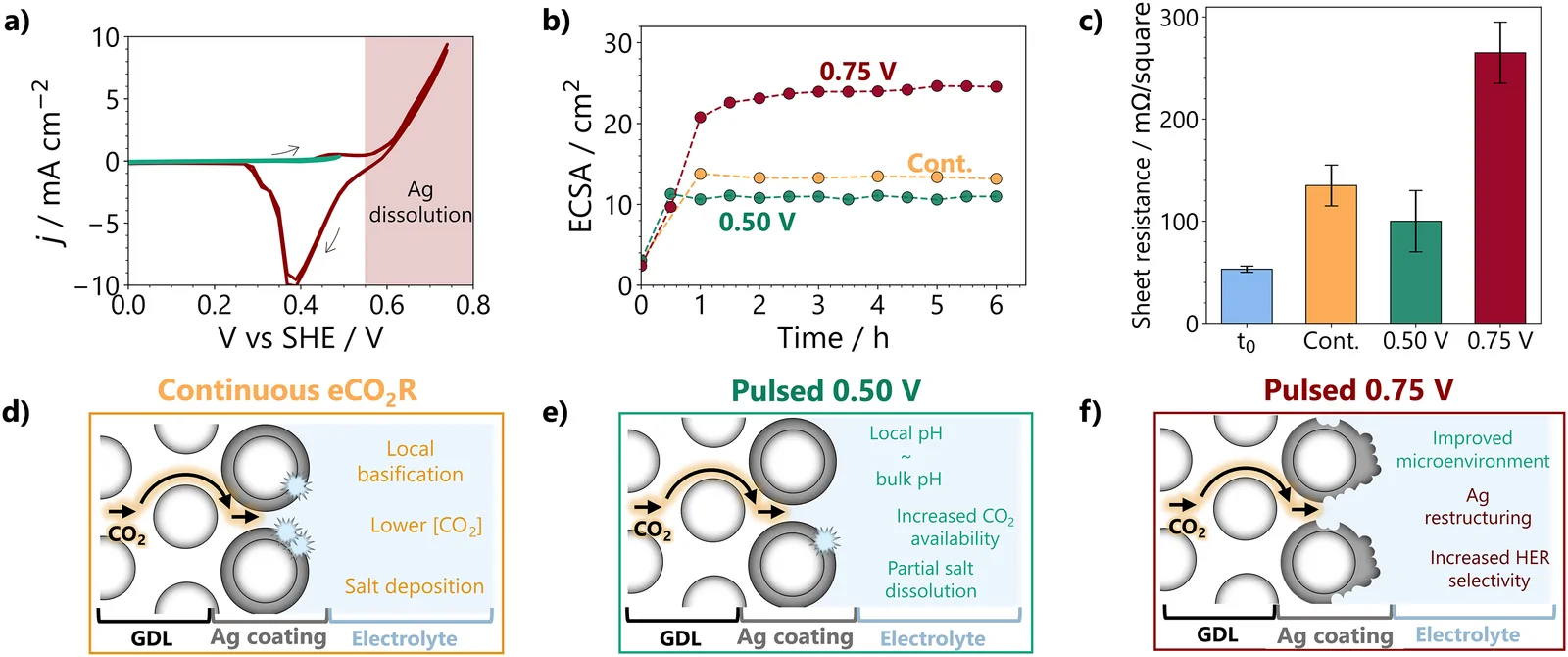
(a) Cyclic voltammetry curves at a scan rate of 20 mV s^−1^ for a silver GDE after 2 hours of continuous electrolysis at 200 mA cm^−2^. (b) ECSA of the silver catalyst measured over time for continuous electrolysis, pulsed electrolysis with 0.50 V applied every 30 minutes, and pulsed electrolysis with 0.75 V applied every 30 minutes. (c) Four-probe sheet resistance for a pristine silver GDE (*t*_0_) and *post mortem* after continuous electrolysis, pulsed electrolysis with 0.50 V applied every 30 minutes, and pulsed electrolysis with 0.75 V applied every 30 minutes. (d)–(f) Schematics illustrating the effect of pulsing on the catalyst microenvironment (d) during continuous electrolysis, (e) during and after a pulse at 0.5 V *vs.* SHE, and (f) during and after a pulse at 0.75 V *vs.* SHE.

The correlation between pulse potential, catalyst restructuring, and selectivity loss suggests that silver dissolution and subsequent redeposition are responsible for the degradation observed under more oxidative pulsing conditions. To validate our hypothesis, we further characterized the electrochemical and structural properties of the silver GDEs. Fig. 3a shows a cyclic voltammogram recorded on a silver GDE after 2 hours of electrolysis at 200 mA cm^−2^. Two distinct features are visible: a sharp anodic current onset at approximately 0.55 V *vs.* SHE corresponding to silver dissolution in a basic environment rich in bicarbonate ions, and a broad cathodic peak around 0.40 V *vs.* SHE attributed to silver replating and surface restructuring.^33,34^ Cyclic voltammetry thus provides a clear explanation for the behavioral difference observed so far: a pulse potential of 0.50 V sits below the anodic dissolution onset, whereas pulses at 0.60 and 0.75 V fall within the silver dissolution region and are followed by a silver redeposition process, consistent with the restructuring and morphological changes of the silver catalyst observed in the SEM images. Direct evidence for pronounced silver dissolution during pulsed electrolysis with 0.75 V pulses is provided by ICP-MS analysis, which measures a silver concentration of 34.5 ± 1.1 µg L^−1^ after 17 hours. In comparison, continuous electrolysis and pulsed electrolysis with 0.50 V pulses result in substantially lower silver concentrations of 1.26 ± 0.18 µg L^−1^ and 0.86 ± 0.05 µg L^−1^, respectively. Despite the enhanced dissolution under 0.75 V pulsing, the total amount of dissolved silver corresponds to less than 1% of the initial catalyst loading. This indicates that the majority of dissolved silver undergoes redeposition, contributing to catalyst surface restructuring.

The evolution of the electrochemically active surface area (ECSA) over time, shown in Fig. 3b, supports this interpretation. Under continuous electrolysis, the ECSA increases from its initial value of 5 cm^2^ and reaches a plateau at around 10 cm^2^, reflecting limited surface roughening under purely reductive conditions. Pulsing at 0.50 V produces a similar ECSA profile, stabilizing at comparable values, consistent with the preserved catalyst morphology observed in Fig. 1e. In contrast, pulsing at 0.75 V drives a rapid and large increase in ECSA, which rises immediately after the first pulse and stabilizes at approximately 24 cm^2^, more than double the value observed under the other two conditions. The large ECSA increase reflects the formation of a highly roughened silver catalyst layer resulting from dissolution and redeposition of silver.^25,28^ We propose that the displacement of silver away from the gas–electrolyte interface and replating in clusters protruding towards the electrolyte, as visible in the cross-sectional SEM image (Fig. 1i and Fig. S6), is a major contributor to the observed selectivity loss. Such a morphology is expected to reduce the number of silver catalytic active sites readily available to gaseous CO_2_ while increasing exposure to the electrolyte phase, favoring HER relative to CO_2_ reduction. Further implications are discussed in Note S4.

The sheet resistance measurements, shown in Fig. 3c, corroborate these structural observations. Relative to the pristine GDE with ∼55 mΩ square^−1^, continuous electrolysis increases the sheet resistance to ∼135 mΩ square^−1^, while pulsing at 0.50 V results in a comparable value of ∼100 mΩ square^−1^. In contrast, pulsing at 0.75 V increases the resistance to ∼265 mΩ square^−1^, consistent with a substantial loss of electrical connectivity within the catalyst layer, likely arising from the fragmentation and redistribution of silver observed by SEM.

Cyclic voltammetry, ECSA evolution, and sheet resistance data converge to a consistent picture in which approximately 0.55 V *vs.* SHE marks the onset of a dissolution-redeposition behavior for silver. Below the dissolution threshold, the improved selectivity retention is consistent with periodic regeneration of the catalyst microenvironment, through local acidification and partial removal of accumulated (bi)carbonate species given the required pulse length of 5–10 seconds (Fig. 3d and e). Above the threshold, the dissolution of silver and redeposition of clusters dominates, irreversibly compromising the catalyst layer and promoting hydrogen evolution (Fig. 3f).

A full picture of the pulse mechanism and related phenomena can realistically only be captured by combining multiple *operando* experiments with high time and spatial resolution and by the introduction of a microkinetic model capturing dynamic electrode wetting, catalyst oxidation state and changes, as well as CO_2_ availability and product formation during pulses. Nonetheless, our work provides experimental evidence of the potential-dependent boundary separating beneficial microenvironment regeneration from detrimental silver dissolution, and highlights the importance of considering catalyst restructuring as a degradation pathway.

## 4. Conclusions

Pulsed eCO_2_R protocols should be carefully selected to improve catalyst lifetime, especially when it comes to oxidative pulse potentials. Below the silver dissolution threshold, brief pulses regenerate the catalyst microenvironment without detectable restructuring of the silver catalyst layer. Above this threshold, pulses trigger dissolution-redeposition cycles that irreversibly restructure the catalyst into a morphology promoting hydrogen evolution. Designing pulsed protocols for silver-based CO_2_ electrolyzers thus requires verification that the chosen oxidative potential falls below the dissolution threshold in the catalyst local environment. Applying an oxidative pulse every 30 minutes with a pulse duration of 10 seconds avoids any catalyst restructuring, providing sufficient time for local electrolyte acidification and (bi)carbonate dissolution. This yields a duty cycle exceeding 99% while sustaining a selectivity in the range of 70–80% over 100 hours of operation, a five-fold improvement in selectivity relative to continuous operation. The brevity of the pulse is advantageous: microenvironmental regeneration is maximized within 10 seconds, avoiding extended eCO_2_R off times. These findings establish that infrequent oxidative pulses, below dissolution threshold, are a practical and energetically efficient route to long-term stability of silver-based CO_2_ electrolyzers.

## Conflicts of interest

The authors declare no conflict of interest.

## Supplementary Material

EY-004-D6EY00140H-s001

## Data Availability

The data that support the findings of this study can be found in a Zenodo repository under the DOI: https://doi.org/10.5281/zenodo.22047184. Supplementary information (SI) contains details on methods, additional electrochemistry, Raman and WAXS experiments and further imaging of the GDEs. See DOI: https://doi.org/10.1039/d6ey00140h.
