# Supplementary material for: Balancing selectivity retention and catalyst restructuring in pulsed CO2 electroreduction on silver
Source: EES Catal. 2026 Aug 21. Online ahead of print. doi: 10.1039/d6ey00140h (PMC13509025; doi:10.1039/d6ey00140h)
Supplement: Supplementary file 1 [file EY-OLF-D6EY00140H-s001.pdf]

Supplementary Information (SI) for EES Catalysis.
This journal is © The Royal Society of Chemistry 2026

Supporting Information

Balancing selectivity retention and catalyst restructuring in pulsed CO_2 electroreduction on silver

Claudia Bissattini Alessi[1,2], Richa Rajadhyax[2], Alessandro Senocrate*[1], Corsin Battaglia[1,2,3]

[1]*Empa - Swiss Federal Laboratories for Materials Science and Technology, Materials for Energy Conversion, CH-8600 Duebendorf, Switzerland*
[2]*Institute of Materials, École Polytechnique Fédérale de Lausanne, CH-1015 Lausanne, Switzerland*
[3]*Department of Information Technology and Electrical Engineering, ETH Zurich, CH-8092 Zurich, Switzerland*
**corresponding author*

Supporting note 1: GDE fabrication and electrochemical set-up

GDEs with silver as catalyst were prepared by DC magnetron sputtering (AJA International Orion) of a ~400 nm silver catalyst layer (sputtering time 10 min, power 5 W m^{-2}, argon pressure 0.004 mbar, deposition rate ~0.6 nm s^{-1} as measured by quartz microbalance (Inficon)) onto one side of an electrospun hydrophobic porous polymer substrate. Polymer substrates were prepared by electrospinning a 15 wt% solution of polyvinylidene fluoride-co-hexafluoropropylene (PVDF-HFP, Merck, Mw ~400k, Mn ~ 130k) with 1 wt% tetraethylammonium bromide (TEABr, , Merck, 98%) in dimethylformamide (DMF, Merck, 227056, 99.8%) at 30 kV, a solution flow rate of 10 µL min^{-1}, collector drum rotation of 400 rpm, and a tip-to-collector distance of 13 cm[1]. Substrate thickness was approximately 100 µm as measured by a micrometer screw gauge. The average pore size was measured by capillary flow porometry (Porolux 1000 Porometer) and was 0.2 µm. Electrical contact was established using conductive aluminum tape, with no exposed tape area in contact with the electrolyte or CO_2 gas.

CO_2 electrolysis was performed in a three-compartment PEEK electrochemical cell (Redox.me, active area 1 cm^2) with separate gas, catholyte, and anolyte chambers, schematized in Figure S1a. The GDE was mounted as the cathode with its hydrophobic polymer side facing the CO_2 gas stream and the Ag-coated side in contact with the catholyte. An IrO_x catalyst electrodeposited onto a perforated Ti foil served as anode, and an Ag/AgCl electrode (stored in saturated KCl) as reference. Catholyte and anolyte chambers were separated by a perfluorosulfonic acid-based cation exchange membrane (FuelCellStore, PFSA D50-U). All experiments used 1 M $KHCO_3$ (99.7%) as electrolyte, pre-saturated with CO_2 by bubbling prior to electrolysis, and 1 M H_2SO_4 (95-97%). The CO_2 flow to the gas compartment was set to 20 mL min^{-1} by a mass flow controller (Bronkhorst) and measured by a flow meter (Bronkhorst). The set-up is schematized in Figure S1b and described in more detail in our previous work[2].

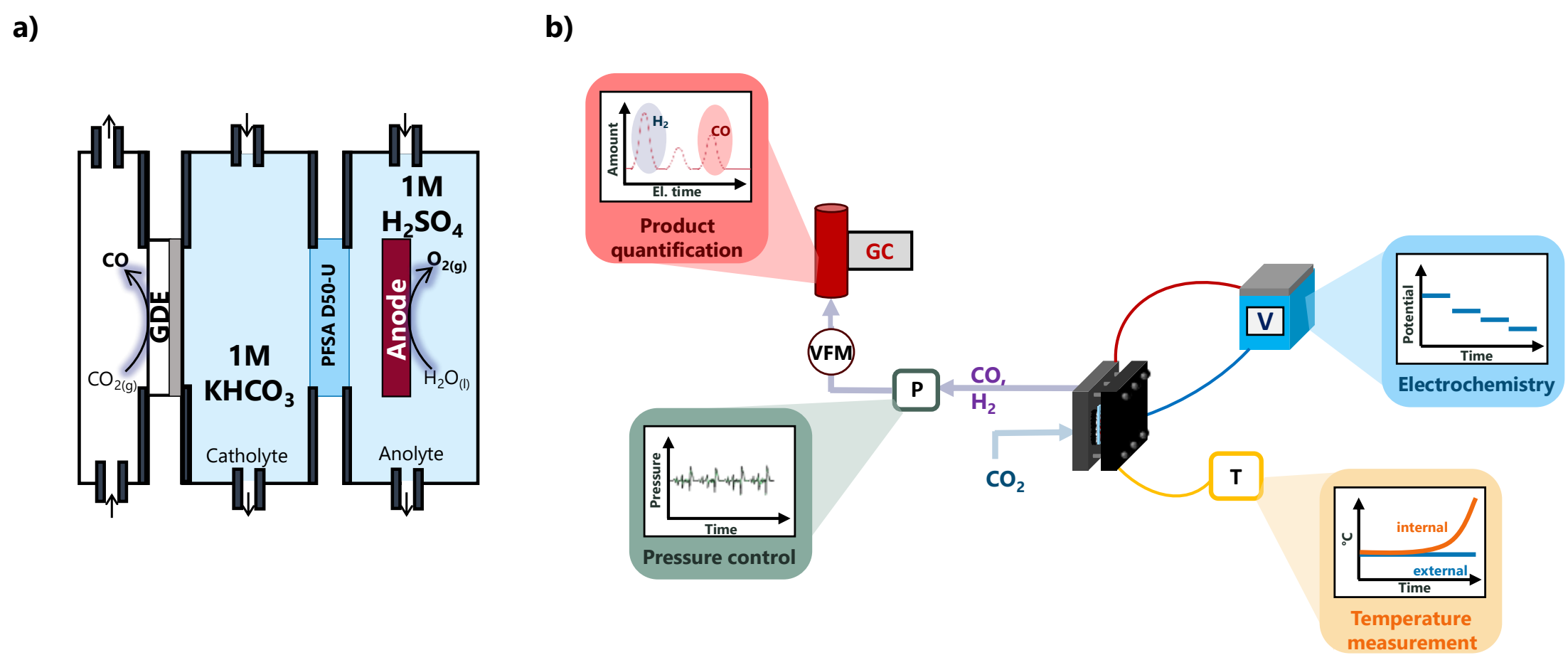

Figure S1: a) Scheme of the three-compartment flow-cell. b) Representation of the set-up sensors and analytics.

The electrochemical protocol applied via a multi-channel potentiostat (Biologic SP300) varied between the different experiments; therefore the specific conditions will be described within the results and discussion section. Electrochemical impedance spectroscopy (EIS) was recorded regularly throughout each experiment to calculate the cell resistance; all reported potentials are iR-corrected and referenced to the standard hydrogen electrode (SHE) scale. Electrochemically active surface areas (ECSAs) were determined by electrochemical double-layer capacitance measurements in the non-Faradaic potential window of 0 to 0.2 V vs SHE at scan rates between 100 and 1000 mV s^{-1}, using an Ag foil as a reference with a specific capacitance of 49.3 ± 1.1 µF cm^2. Gas products were quantified using a two-module micro gas chromatograph (Inficon MicroGC Fusion) equipped with a molecular sieve column (Ar carrier gas) and an RT-Q column (He carrier gas), sampling the gas outlet every 2 min to yield time-resolved faradaic efficiencies over the course of the electrolysis. The gas flow rate was measured at the GC inlet using a volumetric mass flow meter (MesaLabs Defender,). Formic acid accumulated in the catholyte was analysed offline by high-performance liquid chromatography (Agilent HPLC 1260) using a HiPlex-H ion-exchange column with 5 mM H_2SO_4 (98%) as eluent (flow rate 0.6 mL min^{-1}) and a refractive index detector. The samples were diluted 1:1 with 1 M H_2SO_4 to remove the dissolved CO_2 which would result in bubble interference and overpressure in the HPLC. Electrolyte volumes were measured before and after electrolysis using a calibrated variable-volume pipette (Eppendorf) to account for volume changes during operation. As frequent liquid sampling over time can impact selectivity, liquid analysis was only performed at the end for most experiments. It is therefore only used to validate the assumption that all the current that does not produce H_2 is used for eCO_2R . All data concerning formic acid concentration are reported in the Supporting Note 2. The silver catalyst morphology after electrolysis was imaged using scanning electron microscopy (SEM) (Thermo Fisher Phenom). Cross-sectional imaging was performed by focused ion beam scanning electron microscopy (FIB-SEM). A protective carbon layer was deposited on the region of interest using the gas injection system prior to milling. Cross-sections were prepared using a gallium focused ion beam at 30 kV and 15 nA, followed by sequential polishing steps at reduced currents between 3 nA and 700 pA. Secondary electron images were acquired using an Everhart-Thornley detector at 2 kV with currents of 800–1000 pA.

Supporting note 2: Liquid products and dissolved silver quantifications

Table S1 contains the quantified amounts of formic acid in the liquid samples taken at the end (or in some cases during) the experiments discussed in the main text, the corresponding calculated average FE as per Equation S1 (where M is the concentration of formic acid, V is the catholyte volume, F is the Faraday constant and Q is the total charge passed), and the expected amount given the amount of charge remaining after subtracting

the total production of H_2 and CO throughout the experiment. Deviation from the expected amount was on average <2%, so we can assume that the carbon balance is closed.

$$FE_{fa} = \frac{M \cdot V \cdot 2 \cdot F}{Q}$$

Table 1: Amount of formic acid in the catholyte samples for different experiments at different points in time and calculation relative FE.

Experiment	Time / h	Amount / mM	FE_{fa} / %	Expected amount / mM
Figure 3c, continuous	7.2	59	22	54
Figure 3c, continuous	71.7	186	8	186
Figure 3c, pulsed 0.50 V	8.3	75	24	79
Figure 3c, pulsed 0.50 V	30.8	198	24	209
Figure 3c, pulsed 0.50 V	98.9	541	21	540
Figure 3c, pulsed 0.75 V	52	222	11	193

In Table 2 we report the amount of dissolved silver for several of the experiments discussed in this work, as well as an estimate of the catalyst loss relative to the initial amount (0.4 mg) per hour (based on the total length of each experiment). Pulsing at 0.75 V vs SHE introduces a substantial Ag dissolution compared to other protocols, nonetheless the catalyst loss is relatively low.

Table 2: Amount of dissolved silver in the catholyte samples for different experiments.

Experiment	Dissolved Ag / $\mu g\ L^{-1}$	Catalyst loss / ‰ h^{-1}
Figure 1a (continuous)	1.26 ± 0.18	0.02
Figure 1b (0.50 V, 30 min)	0.86 ± 0.05	0.02
Figure 1c (0.75V, 30 min)	34.5 ± 1.1	0.47
Figure S2e (0.60 V, 10 min)	21.1 ± 0.4	0.45
Figure S2l (0.50 V, 120 min)	2.04 ± 0.04	0.02

Supporting note 3: Additional data and reproducibility analysis

Figures S2a-l represent the $FE_{C\text{-}products}$ over time of the experiments out of which the selectivity loss was extracted and the GDE imaged to create Figure 2a. The label left and on top of the figures represent the pulsing potential and pulsing frequency, respectively. Figures S2c and S2k are the same as Figures 1b and 1a.

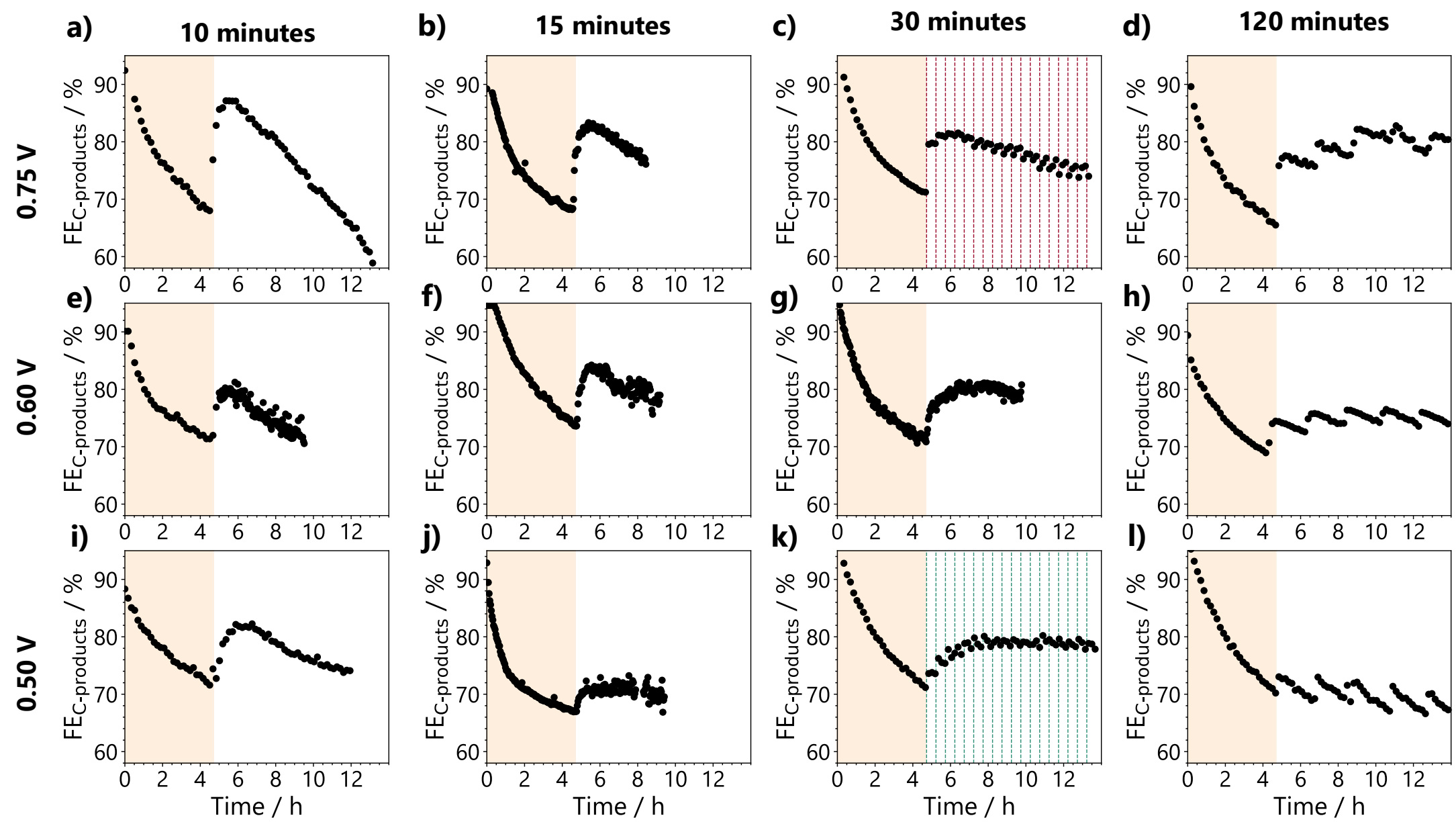

Figure S2: a-l) Raw data ($FE_{C\text{-products}}$ vs time) of the 12 experiments out of which the selectivity loss was extracted for the matrix in Figure 2a.

The first 5 hours of continuous electrolysis of the 12 experiments in Figure S2 were used to assess measurement reproducibility. The individual FE profiles, together with the mean, standard deviation, and 95% confidence intervals, are shown in Figure S3. The experiments exhibited good reproducibility, with an average coefficient of variation (CV) of 4.10% over the entire analysis period. Furthermore, the root-mean-square deviation (RMSE) of individual experiments from the ensemble mean was 2.80 ± 1.41 FE percentage points, indicating that the experimental variability remained small compared with the overall change in FE during electrolysis.

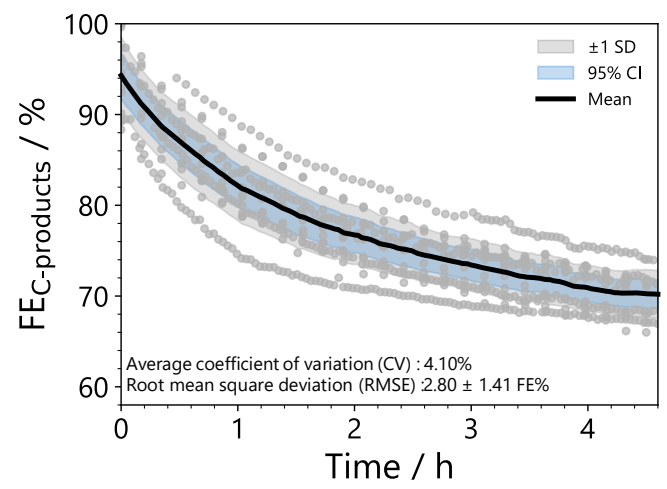

Figure S3: Reproducibility analysis of the continuous electrolysis intervals at the beginning of each experiment.

To assess the reproducibility of the experiment displayed in Figure 2b and verify that at least 5 to 10 seconds at the pulse potential are required to deliver sufficient recovery, we repeated the experiment starting from higher pulse times (20 seconds) down to 1-second pulses, and then back to 5-second pulses. The results are shown in Figure S4. Similarly to the experiment discussed in the main text, 20 to 5 seconds pulses allow for a total recovery of the lost FE. Once the pulse time is lowered further the selectivity starts to drop again and is only recovered upon switching back to 5-second pulses.

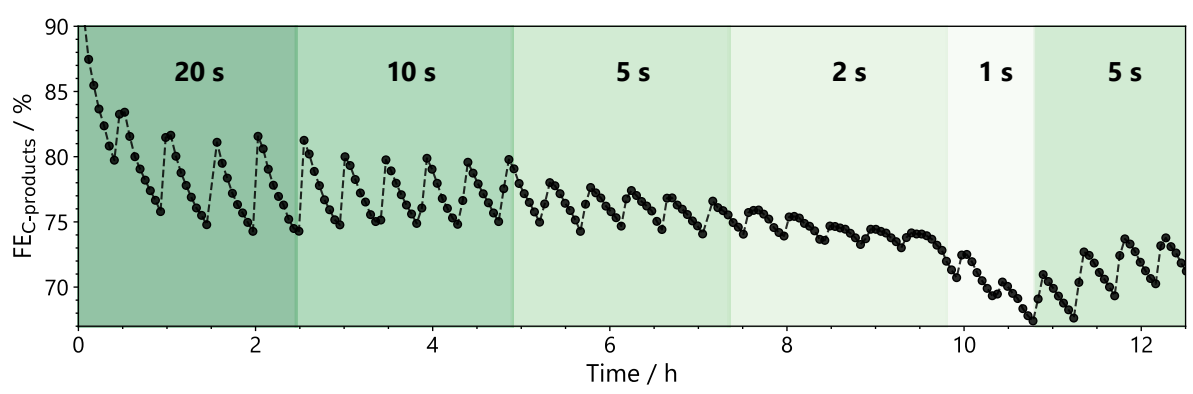

Figure S4: Faradaic efficiency towards carbon products for different oxidative pulse durations with pulses at 0.50 V applied every 30 minutes.

Given the stable and high performance demonstrated by the long-term pulsed electrolysis experiment with 0.75 V pulses in the first 10 hours (described in the main text and shown in Figures 3c and S5), we investigated whether a minor restructuring could indeed be beneficial for the catalyst and lead to continued high FE once switched to 0.50 V pulses. The experiment shown in black in Figure S5, shows that this is not the case. Once the pulsing regime switches from 0.75 V to 0.50 V, the FE drops and approximates the plateau of the experiment in which 0.50 V pulses were applied from the start. We can thus conclude that the restructuring is not providing any lasting improvement to the catalyst. A hypothesis for the higher average FE could be that the dissolution of the silver itself is cleaning the surface of precipitates better than can be achieved with the lower voltage pulses.

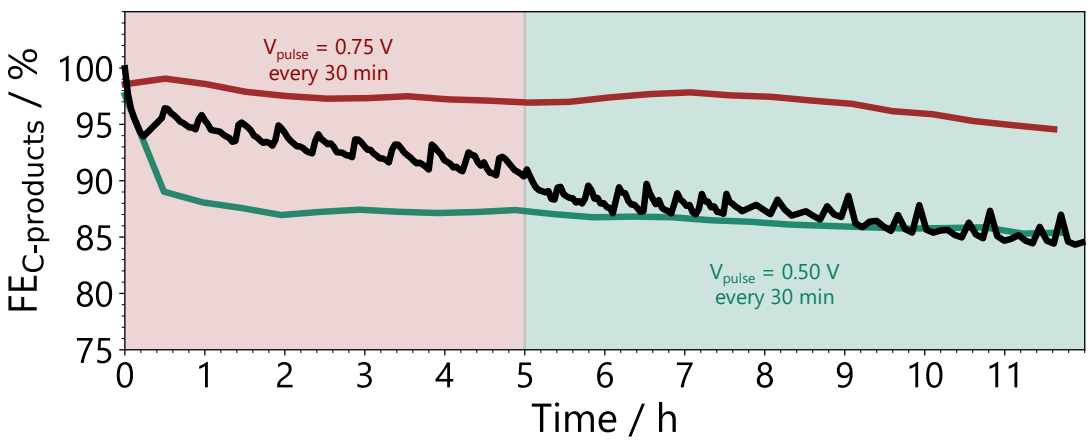

Figure S5: Faradaic efficiency towards carbon products for 12 hours of pulsed electrolysis with 10s - 0.75 V oxidative pulses every 30 minutes for 5 hours followed by pulsed electrolysis with 10s - 0.50 V oxidative pulses every 30 minutes, compared to the evolution of the faradaic efficiency during pulsed electrolysis with either 0.75 V or 0.50 V oxidative pulses for 10 seconds every 30 minutes (taken from Figure 2c in the main text).

Supporting note 4: Silver restructuring on different GDEs

The silver clusters formed during pulsed operation are not uniformly distributed but appear as macroscopic stripes across the GDE surface (Figure S6a-b-c). We attribute this behavior to the repeated amplification of pre-existing current-density heterogeneities within the fibrous electrode architecture[3,4]. After each oxidative pulse, regions experiencing slightly higher local current densities serve as preferred sites for redeposition. Repetition of this dissolution-restructuring cycle over hundreds of pulses progressively amplifies these spatial differences, leading to the observed striped morphology. Therefore, some regions end up with a lower Ag availability at the gas-liquid interface, while others present agglomerates protruding toward the electrolyte, as can be seen clearly in Figure S6b. In contrast, during cyclic voltammetry (Figure S6d) the dissolution and redeposition processes occur during a single continuous potential sweep, limiting the cumulative amplification of local current heterogeneities and resulting in a more uniform distribution of silver clusters, causing nonetheless selectivity loss over time.

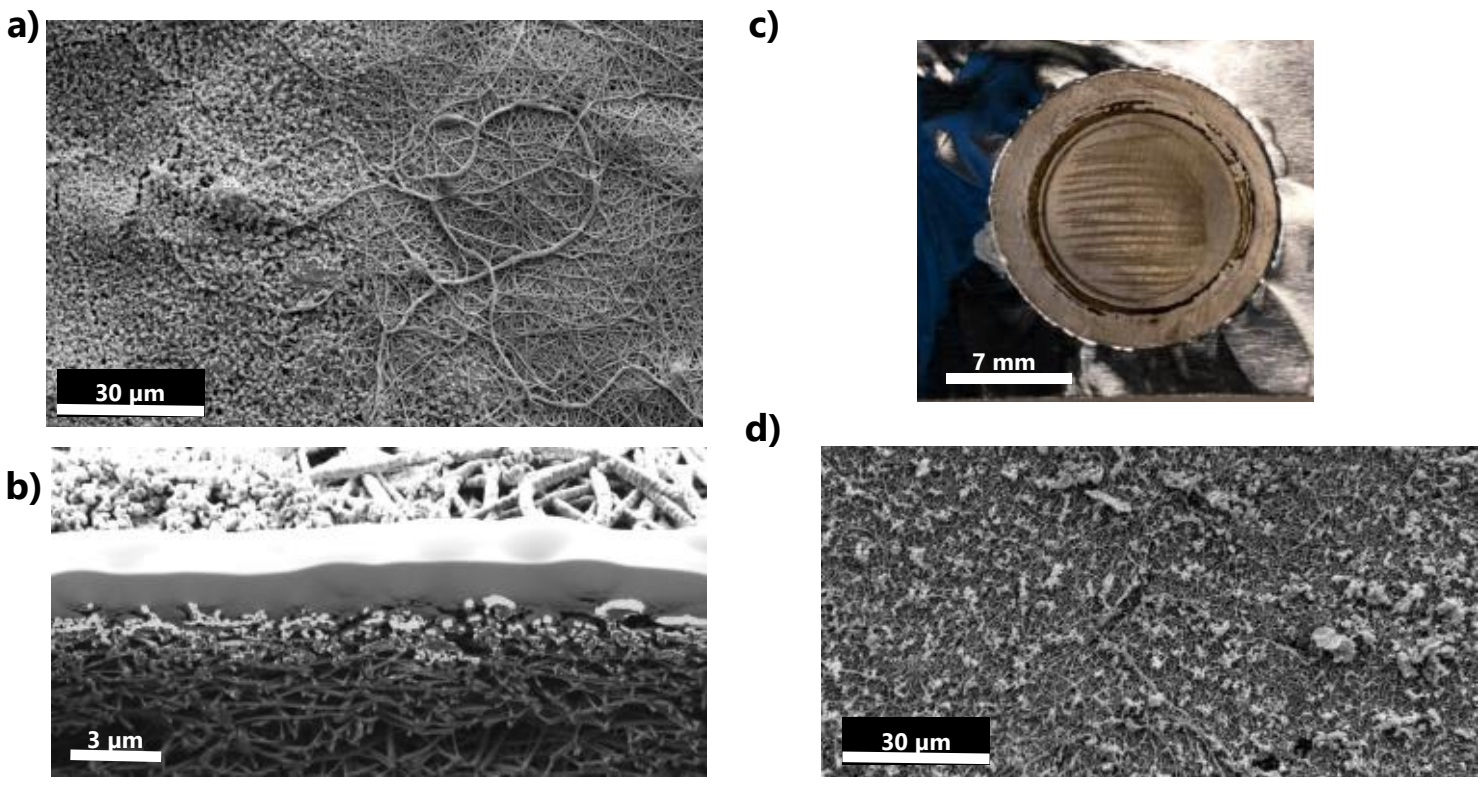

Figure S6: SEM image of the a) top view and b) cross-section of a silver GDE after pulsed electrolysis with 0.75 V pulses at the intersection between a dark and a light stripe of the surface c) Photography of the silver coated side of the silver

GDE after pulsed electrolysis with 0.75 V pulses. d) SEM image of the top view of a silver GDE after multiple cyclic voltammetry cycles between 0.75 V and -4.2 V.

Figure S7 shows the appearance of the GDEs after the three long-term experiments in Figure 2c. After 72 hours of continuous electrolysis, there was no significant difference compared to shorter protocols (see Figure S7a vs Figure 1d). After 100 hours of pulsing at 0.50 V, the surface of the silver coated fibers appeared consistently rougher than in the continuous case, suggesting some mild restructuring driven by the pulses (Figure S7b). After 52 hours pulsing at 0.75 V, there were multiple agglomerates of different sizes and micrometer-wide tears in the previously uniform Ag coating of the GDE (see Figure S7c).

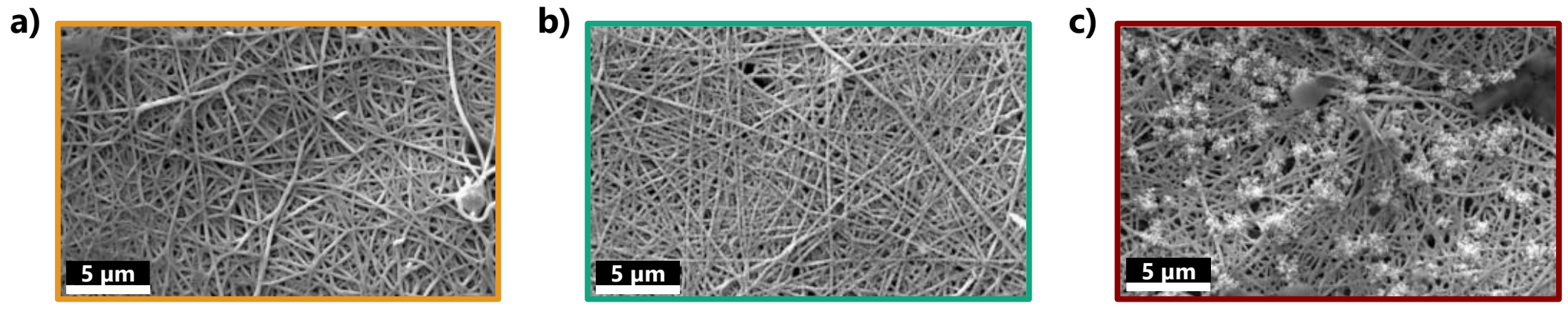

Figure S7: a-c) SEM image of the top view of a silver GDE after the long-term experiments in Figure 2c.

Figure S8 is discussed in the main text. In Figure S8a, we report the almost total isolation of the active area from the rest of the GDE and current collector after the 52 hours of 0.75 V pulsed electrolysis. This is likely caused by mechanical stress stemming from periodic uneven restructuring. The evolution of the working electrode potential over time for the three long-term experiments in Figure 3c is reported in Figure S8b: notably, the continuous and the 0.50 V experiments had a stable potential, while the potential for the 0.75 V follows a trend similar to its faradaic efficiency.

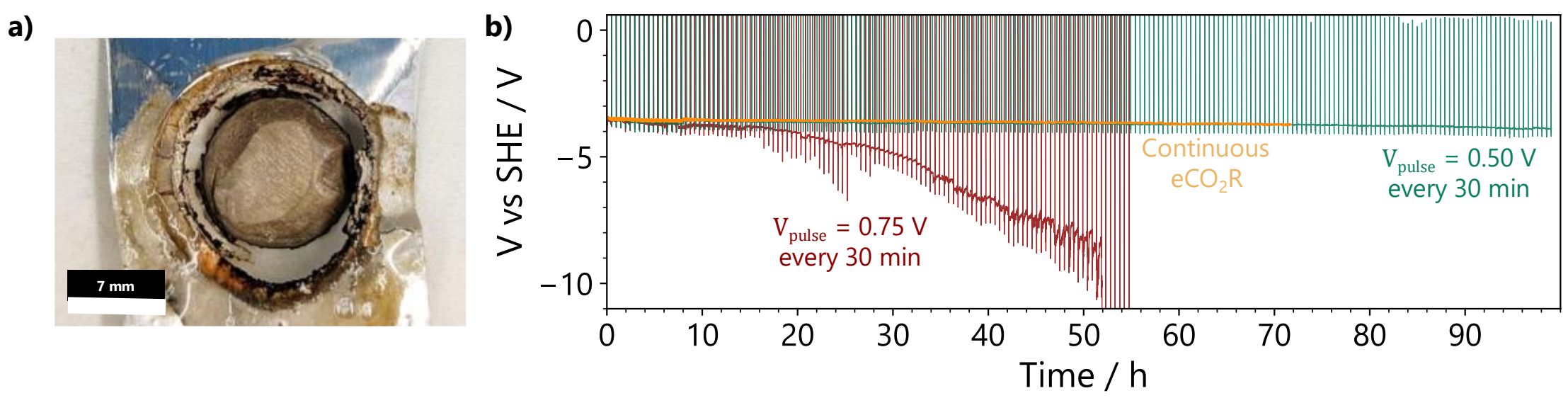

Figure S8: a) Photo of the silver GDE after 52 hours of pulsed electrolysis at 0.75 V. b) Evolution of the working electrode potential during the three long-term experiments.

Given the potential dependence of Ag dissolution, we expect the identified 0.55 V threshold for beneficial pulsing to be similar for polymer- and for carbon-based substrates, since it is set by silver electrochemistry rather than by the gas diffusion layer composition. We conducted an experiment using an AvCarb GDS2230 gas diffusion layer sputtered with Ag. Due to their optimized microporous layer, these GDEs are less sensitive to CO_2 transport limitations at -200 mA cm^{-2}, with very slow degradation and Faradaic efficiencies too close to 100% to require pulsing. To achieve a better comparison to the polymer GDEs we thus increased the current density to -300 mA cm^{-2} and we alternated between continuous, pulses at 0.50 V and pulses at 0.75 V with the same unflooded sample. As shown in Figure S9a, the pulses provide a 3-5% recovery, similar to that of the polymer GDEs (Figure 2b).

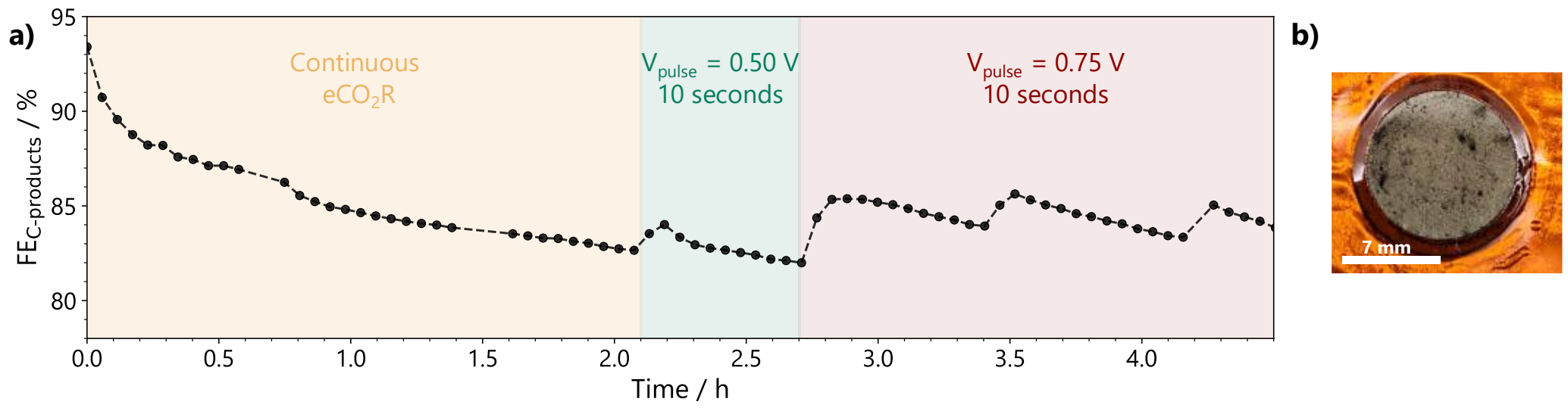

Figure S9: a) Faradaic efficiency towards carbon products for a carbon-based GDE for different pulsing protocols at -300 mA cm^{-2}. b) Photo of the GDE at the end of the experiment.

The pulses at higher voltage seem to raise the average FE, however, as can be assumed from the darker regions on the *post-mortem* GDE's surface in Figure S9b, catalyst restructuring may end up degrading the performance also for these substrates. It is interesting to notice the different pattern on the surface of the carbon GDE compared to the polymer. While the fibers of the polymer determined a specific "stripe-like" pattern of restructuring, we expect that the unevenness of the current distribution in these substrates will be tied to the underlying microporous layer, where clusters of carbon black may be present in certain areas.

Supporting note 5: Further considerations on mechanisms

We collected additional evidence to validate the selectivity recovery mechanisms proposed in the main text, combining a kinetic analysis of the relevant timescales, operando Raman spectroscopy of local (bi)carbonate speciation, and a control experiment in which the oxidative pulse is replaced by an open circuit hold.

The recovery observed after each pulse develops over one to ten seconds and saturates beyond ten seconds. Several processes could in principle occur on this timescale. Electrochemical double layer relaxation, $\tau_{DL} = R_{ct} \cdot C_{dl}$, assuming a double-layer capacitance of C_{dl} = 40 µF cm^{-2} and taking an average of charge transfer resistance from impedance measurements R_{ct} = 1.5 Ω cm^2 for electrolyte contacted metal electrodes, gives τ_{DL} of 60 µs, several orders of magnitude faster than the observed window[5]. Oxidative stripping of adsorbed intermediates requires a monolayer charge of 200 to 400 µC cm^{-2}, delivered within 0.2 to 0.4 s at a pulse current density of ~ 1 mA cm^{-2} used here[6]. Therefore, we excluded these two mechanisms from our analysis.

We looked for direct evidence of a local pH and speciation change using operando surface-enhanced Raman spectroscopy (SERS) in the dedicated cell represented in Figure S10. The ratio of the area of HCO_3^- and CO_3^{2-} peaks has previously been used to estimate local pH near CO_2 reduction electrodes and we apply the same approach here[7–9]. The enhancement in our measurement comes from the sputtered Ag catalyst layer itself, which is SERS-active[10]. The symmetric stretch of HCO_3^- appears near 1015 cm^{-1} and that of CO_3^{2-} near 1066 cm^{-1}. The relative intensity of these bands reflects the local HCO_3^- / CO_3^{2-} ratio, which is set by local pH through the second dissociation equilibrium of carbonic acid, so this ratio can be converted into a local pH estimate once referenced to a known bulk value[8]. The method reports an average pH over the SERS-active probed volume rather than a value strictly at a single point on the catalyst and the resulting spectra will also present Raman peaks related to the gas diffusion layer.

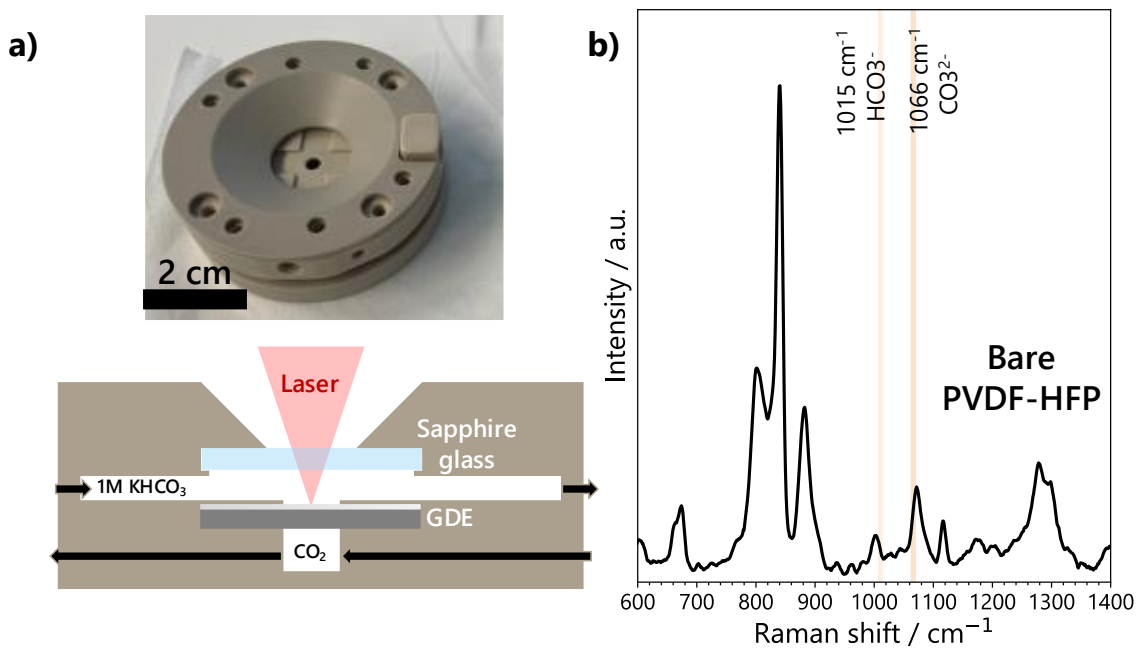

Figure S10: a) *Operando* Raman cell: photo and scheme. b) Raman spectra of bare PVDF-HFP (20x, 120 seconds, 50 LP, 1 acc)

The Raman bands of our standard PVDF-HFP substrate overlap the carbonate region, as visible in the spectra of the bare polymer shown in Figure S10b, making polymer substrate non ideal for these experiments. We therefore used a carbon paper gas diffusion layer (AvCarb GDS2230) sputtered with Ag under the same conditions as our standard GDEs. Electrolysis was carried out in 1 M $KHCO_3$, recirculated at 5 mL min^{-1}, with a CO_2 flow of 5 mL min^{-1} and an active area of 0.13 cm^2. The cell was mounted under a Renishaw Invia Reflex Raman spectrometer and imaged through a sapphire window with a 20x objective. Spectra were collected with a 785 nm laser, 1200 lines mm^{-1} grating, 50% laser power, 20 s acquisition, and 4 accumulations. The potential was stepped in 100 s intervals between -1.3 V and 0.3 V vs SHE, with each spectrum acquired 10 s after each step, and additional spectra collected at open circuit voltage (OCV) before and after the sequence. The analysis is restricted to this potential range as above 0.4 V Ag oxidation to Ag_2O introduces a strong band near 1058 cm^{-1} that overlaps the CO_3^{2-} band[11]. Peak areas were obtained by fitting the Raman spectra with Gaussian line shapes after linear baseline subtraction in the 950 to 1120 cm^{-1} region (Figure S11c, top panel). The peak near 1080 cm^{-1} was assigned to CO_3^{2-} adsorbed on the silver surface[7,12]. This adsorbed component had to be fit to disentangle the partially overlapping dissolved CO_3^{2-} peak but was excluded from the pH ratio, since the Henderson-Hasselbalch relationship applies to the aqueous acid-base equilibrium between HCO_3^- and CO_3^{2-}, while the adsorbed species is governed by a surface equilibrium dependent on potential and coverage rather than pH[9]. Peak position, width, and area were allowed to vary freely within 5 cm^{-1} of each expected position, and integrated area was taken as proportional to concentration. The fraction of HCO_3^- relative to total dissolved carbonate was calculated at each potential from the two aqueous-species areas (Figure S11c, bottom panel). Taking the independently measured bulk pH of 7.8 as the local pH at OCV, the fraction of HCO_3^- at -1.3 V, corresponding to -15 mA cm^{-2}, converts to a local pH of 8.6. Higher current densities could not be reached because gas evolution at the surface scattered the laser. This pH increase of 0.8 units is consistent in sign and order of magnitude with the alkalinization predicted above. We note that since the two experiments differ in current density and GDE architecture, this approach only provides a qualitative confirmation. At 0.3 V vs SHE, the fraction of HCO_3^- returned to a value comparable to OCV, indicating that a mildly positive potential is sufficient to restore the local pH within the timescale probed. Ag_2O formation prevents this measurement above 0.4 V, but since Ag oxidation releases protons ($2Ag + H_2O \rightarrow Ag_2O + 2H^+ + 2e^-$), the local pH at the pulse potentials used in the main text (0.50 to 0.75 V) is expected to be at or even below the value measured at 0.3 V[13].

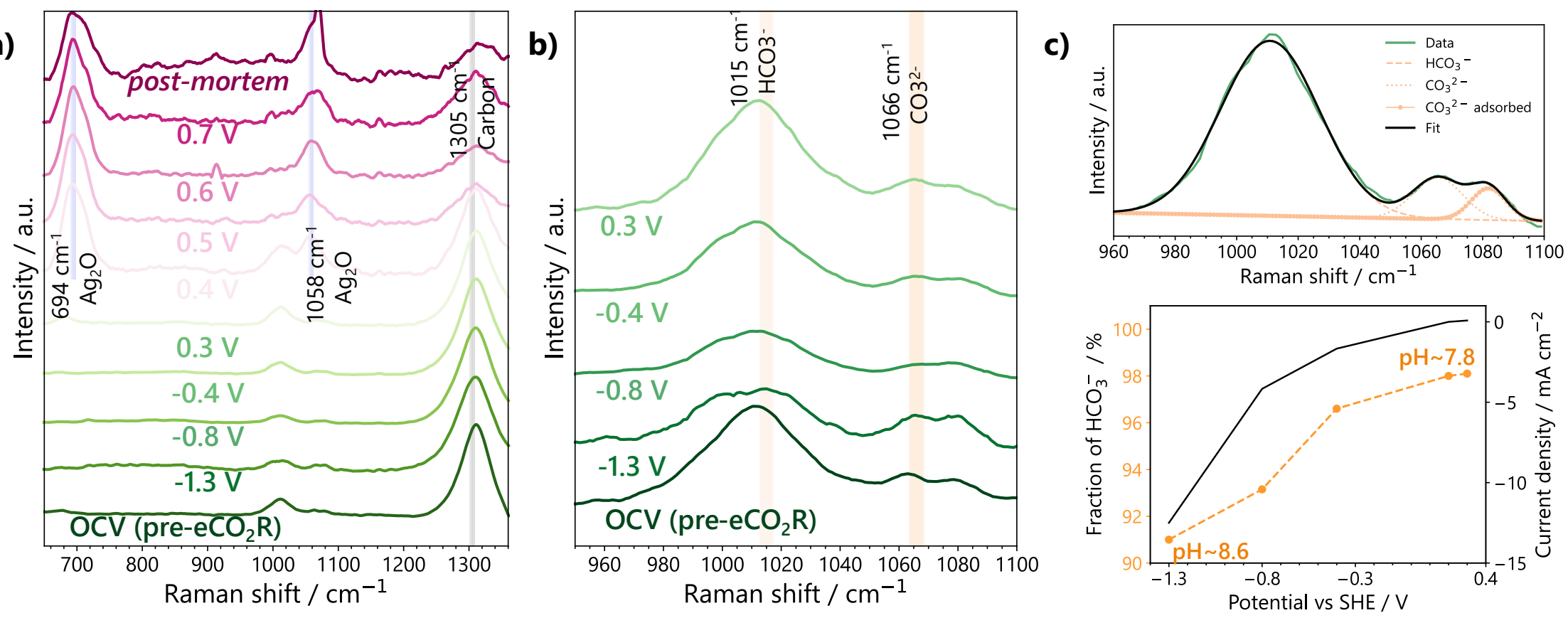

Figure S11: *Operando* Raman measurements at the Ag catalyst surface at different potentials. a) Measurements collected for 80 seconds after 10 seconds at given potential (-1.3 to 0.7 V vs SHE) and at OCV in the Raman cell before and after the electrochemical sequence. b) Detail of the same spectra between -1.3 V and 0.3 V around the peaks related to (bi)carbonate species. c) Gaussian fit of the peaks used to estimate the local pH (up) and fraction of bicarbonate ion and recorded current density at different potentials (down).

To determine whether Ag oxidation itself, rather than the accompanying change in local chemistry, was responsible for the recovery, we replaced the 0.50 V oxidative pulse with a hold at open circuit voltage. In the experiment shown in Figure S12, we alternated OCV pulses of different durations between 2 and 20 seconds to compare the results to those of the experiment in Figure 2b. Again, increasing the pulse duration to 10 seconds led to more stable and higher FE.

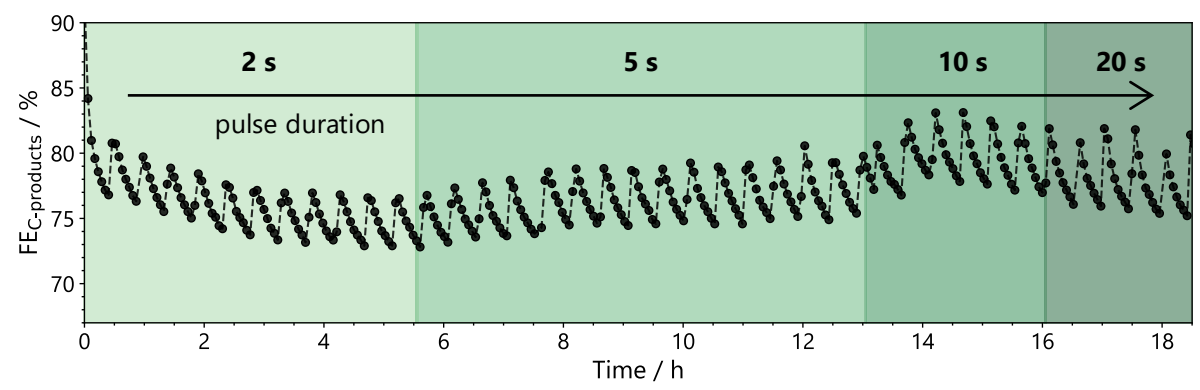

Figure S12: Faradaic efficiency towards carbon products for different pulse durations with pulses at OCV applied every 30 minutes.

This result excludes Ag oxidation and re-reduction or any modification of the silver structure as the operative recovery mechanism. The similarity in the characteristic duration needed for recovery under OCV and under the oxidative pulse further indicates that the same process is rate limiting in both cases: interrupting the reductive current is enough to recover most of the selectivity, without additional proton generation at the interface, and the bulk electrolyte and gas phase CO_2 are sufficient to drive the local re-equilibration.

However, these considerations still do not explain why a degradation that develops over roughly 30 minutes can recover within about 10 seconds. The kinetics of CO_2 hydration/dehydration are expected to be similar in both directions and cannot alone account for this[14]. On the contrary, salt precipitation and dissolution happen on different timescales. Nucleation and growth from solution are inherently slow and are typically detectable within a timescale of tens of minutes[15,16]. Meanwhile dissolution of an already-formed, thin deposit is comparatively fast once local undersaturation is reached[17]. We therefore propose that the local pH swing given by the pulse redissolves a fraction of this precipitate within seconds. This connection is inferred from the timescale asymmetry and literature report, but represents the most likely mechanism and is in agreement with the current evidence.

We ran an *operando* wide-angle X-ray scattering experiment on the electrospun PVDF-HFP GDEs coated with Cu instead of Ag and with a 2 M KCl electrolyte (as a $KHCO_3$ electrolyte would not have allowed to distinguish local bicarbonate salt precipitation from electrolyte drying). The experimental setup and the alignment procedure for the operando WAXS experiments were reported in our previous work[15]. While we recognize that these operating conditions, particularly the different catalyst, may not fully represent the experiments discussed in this work, we provide this data to show the importance of precipitated bicarbonate salts in polymer-based as degradation mechanism. Figure S13 shows the $FE_{C\text{-products}}$ and the evolution of the intensity of the main diffraction peak associated with $KHCO_3$ (found at q = 0.87 Å^{-1}), averaged over the entire GDE, over the course of a continuous experiment and one with 10-second pulses at 0.30 V vs SHE every 10 minutes. No clear trends or differences emerge between the two regimes. As $KHCO_3$ was detected under active pulsing while retaining selectivity, we can infer that only a locally relevant fraction of the precipitate needs to be cleared to restore selectivity.

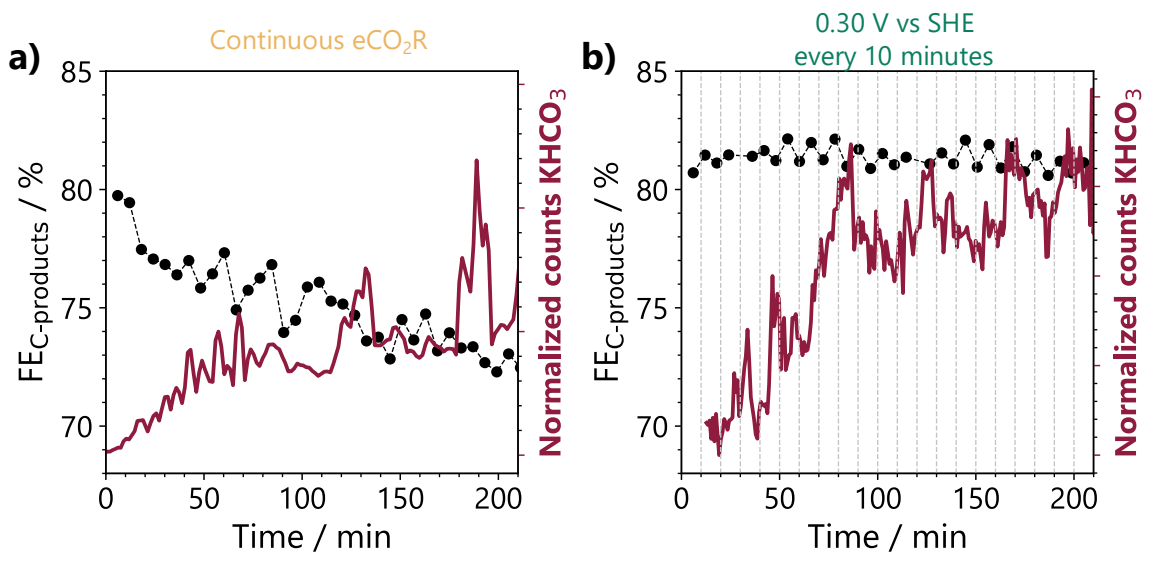

Figure S13: Faradaic efficiency towards carbon products and intensity of the peak at q = 0.87 Å^{-1} for a) continuous electrolysis at -200 mA cm^{-2} and b) pulsed electrolysis with 0.30 V pulses every 10 minutes. The intensity of the peak associated with $KHCO_3$ is calculated by subtracting the background signal at 0.82 Å^{-1}, where no peak is present, from the most intense signal in the region 0.83-0.87 Å^{-1}.

Supporting note 6: Impact of the operating current density

Figure S14 addresses the effect of operating current density, plotting $FE_{C\text{-products}}$ normalized to its initial value as a function of cumulative charge passed, for current densities of 100, 200, and 400 mA cm^{-2}. Under continuous electrolysis, all three current densities show a clear decay in normalized $FE_{C\text{-products}}$, with higher current densities degrading more rapidly on an absolute time basis. When expressed against cumulative charge, however, the decay profiles at different current densities converge closely, indicating that degradation is primarily driven by the total charge passed rather than by the current density itself. Upon introduction of pulsing, all three current densities recover and stabilize at similar normalized $FE_{C\text{-products}}$ values of approximately 90-95% of their initial performance, demonstrating that the pulsing strategy is effective across a wide range of operating current densities. This charge-based equivalence has a practical implication: by reducing the time between pulses proportionally to the increase in current density, equivalent long-term performance can be maintained at higher current densities.

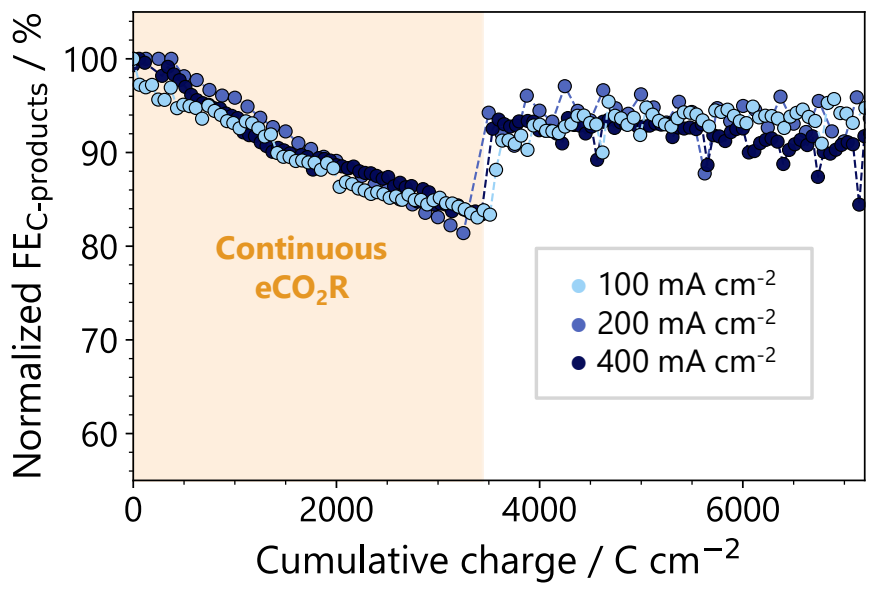

Figure S14: FE$_{C\text{-products}}$ relative to the initial FE$_{C\text{-products}}$ value over cumulative charge passed for different current density values.

Bibliography

1 F. Bernasconi, A. Senocrate, P. Kraus and C. Battaglia, *EES Catalysis*, 2023, **1**, 1009–1016.

2 A. Senocrate, F. Bernasconi, P. Kraus, N. Plainpan, J. Trafkowski, F. Tolle, T. Weber, U. Sauter and C. Battaglia, *Nat Catal*, 2024, **7**, 742–752.

3 A. L. Clemens, J. T. Davis, C. A. Orme, A. Ivanovskaya, R. Akolkar and N. A. Dudukovic, *J. Electrochem. Soc.*, 2023, **170**, 012502.

4 X. Wei, M. Noyong and U. Simon, *iScience*, 2024, **27**, 111119.

5 S. Trasatti and O. A. Petrii, *Journal of Electroanalytical Chemistry*, 1992, **327**, 353–376.

6 B. E. Conway, *Electrochemical Supercapacitors*, Springer US, Boston, MA, 1999.

7 S. Jiang, K. Klingan, C. Pasquini and H. Dau, *J. Chem. Phys.*, 2018, **150**, 041718.

8 Z. Zhang, L. Melo, R. P. Jansonius, F. Habibzadeh, E. R. Grant and C. P. Berlinguette, *ACS Energy Lett.*, 2020, **5**, 3101–3107.

9 W. W. Rudolph, G. Irmer and E. Königsberger, *Dalton Trans.*, 2008, **37**, 900–908.

10 E. L. Ru and P. Etchegoin, *Principles of Surface-Enhanced Raman Spectroscopy: and Related Plasmonic Effects*, Elsevier, 2008.

11 P. Berastegui, C.-W. Tai and M. Valvo, *Journal of Power Sources*, 2018, **401**, 386–396.

12 H. An, L. Wu, L. D. B. Mandemaker, S. Yang, J. de Ruiter, J. H. J. Wijten, J. C. L. Janssens, T. Hartman, W. van der Stam and B. M. Weckhuysen, *Angewandte Chemie International Edition*, 2021, **60**, 16576–16584.

13 P. Delahay, M. Pourbaix and P. V. Rysselberghe, *J. Electrochem. Soc.*, 1951, **98**, 65.

14 K. S. Johnson, *Limnology and Oceanography*, 1982, **27**, 849–855.

15 F. Bernasconi, N. Plainpan, M. Mirolo, Q. Wang, P. Zeng, C. Battaglia and A. Senocrate, *ACS Catal.*, 2024, **14**, 8232–8237.

16 M. Sassenburg, M. Kelly, S. Subramanian, W. A. Smith and T. Burdyny, *ACS Energy Lett.*, 2023, **8**, 321–331.

17 J. W. Mullin, *Crystallization*, Elsevier, 2001.
